# Evaluation of tunica vaginalis flap-covering combined with modified Glenn–Anderson in one-stage repair of proximal hypospadias with incomplete penoscrotal transposition

**DOI:** 10.3389/fped.2022.872027

**Published:** 2022-12-13

**Authors:** Xin Wang, Yong Guan, Yong Wu, Cong Wang, Xiong Ma, Zhenhua Zhang, Dongzheng Zhang

**Affiliations:** Department of Pediatric Surgery, Tianjin Children's Hospital, Tianjin, PR China

**Keywords:** proximal hypospadias, penoscrotal transposition, one-stage repair, value of tunica vaginalis flap, modified glenn anderson

## Abstract

**Objective:**

To explore a novel repair method for proximal hypospadias with incomplete penoscrotal transposition in children and evaluate its safety and outcomes.

**Methods:**

A retrospective analysis of clinical data was conducted for 86 children with severe proximal hypospadias with incomplete penoscrotal transposition who were hospitalized in our department between June 2018 and February 2021. In total, 42 patients (Group A) underwent repair following a one-stage method in which tunica vaginalis flap-covering was combined with a modified Glenn–Anderson procedure, while 44 patients (Group B) underwent a two-step repair consisting of tunica vaginalis flap-covering using the Duplay technique and the modified Glenn–Anderson procedure. The two groups were compared on operation time, length of postoperative hospital stay, postoperative complications, and associated costs.

**Results:**

All operations were successful in both groups. No statistical difference was observed between the two groups in incidence of stenosis of the urinary meatus (2.38% vs. 4.54%, *P* = 0.279), urethral stricture (2.38% vs. 2.27%, *P* = 0.948), urinary fistula (7.14% vs. 6.82%, *P* = 0.907), or urinary infection (7.14% vs. 4.55%, *P* = 0.309). Additionally, there was no statistical difference between the groups in operation time (63.21 ± 5.20 vs. 62.07 ± 4.47 min, *P* = 0.059), postoperative off-bed time (7.02 ± 1.32 vs. 6.84 ± 1.20 days, *P* = 0.456), or duration of hospitalization (10.55 ± 1.15 vs. 10.15 ± 1.45 days, *P* = 0.092). However, Group B patients underwent an additional second-stage operation, incurring extra costs. Three months after surgery, Group A were judged more positively on the PPPS (specifically receiving higher scores on shaft skin and general appearance) by both the parents (shaft skin: 2.10 ± 0.82 vs. 1.93 ± 0.62, *P* = 0.024; general appearance: 2.16 ± 0.91 vs. 1.93 ± 0.72, *P* = 0.042) and the surgeon (shaft skin: 2.42 ± 0.70 vs. 2.25 ± 0.58, *P* = 0.025; general appearance: 2.38 ± 0.69 vs. 2.29 ± 0.51, *P* = 0.041). In most cases, the parents and surgeon were satisfied with the appearance of the genitals after one-stage repair.

**Conclusion:**

The advantages of the novel repair technique include use of a single-stage operation, producing a better appearance at a lower cost. The tunica vaginalis flap-covering method is not only demonstrated to be safe and effective, but it is also a simpler method than the conventional operation.

## Introduction

Hypospadias is a common congenital condition, occurring in about 9.3 per 10,000 live births in China, with an upward trend in incidence (1). It is characterized by abnormal positioning of the urethral orifice, with proximal hypospadias being identified in 25% of cases. The proximal type usually has a higher incidence of complications (2). In cases of proximal hypospadias, penoscrotal transposition makes the repair extremely challenging. The more common form of this is incomplete penoscrotal transposition, in which the penis lies in the middle of the scrotum. Various methods for surgical correction of incomplete penoscrotal transposition have been described, with a modified Glenn–Anderson method being commonly used (3). Two-stage repairs for severe proximal hypospadias are safer and simpler (2); these include use of Bracka's or Byars' as the first stage of the procedure and the Duplay technique as the second stage (4). Subsequently, the modified Glenn–Anderson technique can be employed as a third stage for repair of incomplete penoscrotal transposition (5). During this course of treatment, patients must undergo multiple painful procedures for both surgery and other aspects of care. Furthermore, multi-stage procedures are invariably linked to a greater incidence of surgical complications and higher health care costs. Here we present a new modified technique combining Glenn–Anderson with tunica vaginalis flap-covering using the Duplay technique in a one-stage repair for proximal hypospadias with penoscrotal transposition.

## Materials and methods

**Figure 1:**
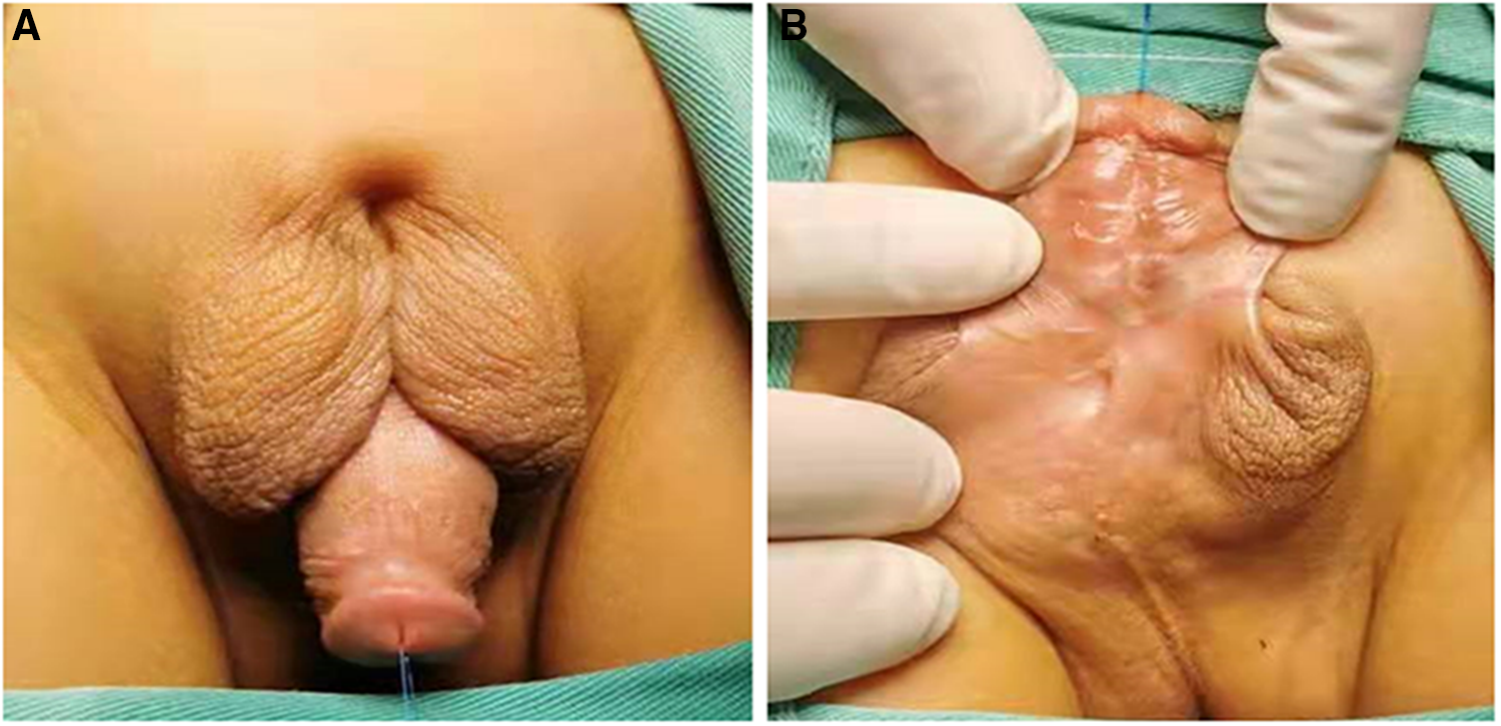
The appearance of proximal hypospadias with incomplete penoscrotal transposition. (**A**) Dorsal view; (**B**) Ventral view.

Our retrospective study enrolled 86 patients with proximal hypospadias with incomplete penoscrotal transposition who underwent surgical treatment in the urology department of Tianjin Children's Hospital from June 2018 to February 2021. These patients needed urethroplasty and correction of penoscrotal transposition. All patients had typical chromosomes (46XY). By February 2022, the average follow-up period was 24.39 ± 4.72 months (range: 12–32 months). We confirmed all patient records by querying both the registration system of the medical records department and the electronic medical records system of Tianjin Children's Hospital. The mean age across all patients was 36.96 ± 21.22 months (6–97 months). The mean age of Group A patients was 36.52 ± 21.63months (6–86 months), and that of Group B patients was 37.36 ± 21.05 months (8–97 months). All patients presented with severe proximal hypospadias with incomplete penoscrotal transposition (Figure 1). For the first stage of surgical treatment, Byars' approach was employed in all patients to reposition the foreskin ventrally, preserving the urethral plate, and straighten the penis. For the second stage, 42 patients (Group A) underwent repair in the form of a one-step method combining the tunica vaginalis flap-covering Duplay technique and the modified Glenn–Anderson, while the other 44 patients (Group B) underwent two-step repair. All operations were performed by the same doctor (Dr Guan).

### Surgical technique

Byars' approach was adopted, the prepuce repositioned ventrally, and the penis straightened for all patients.

#### Group A

**Figure 2:**
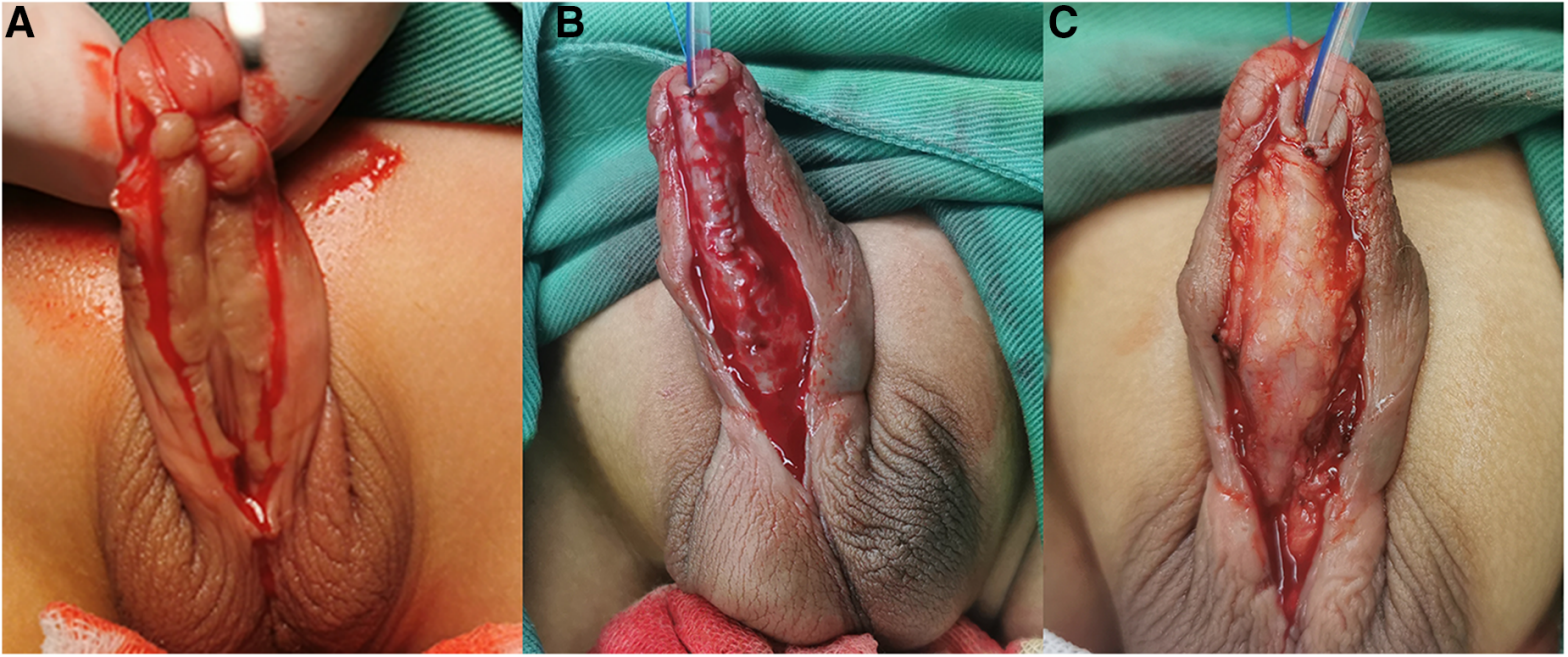
(**A**) A U-shaped skin incision was cut with its base proximal to the urinary meatus. (**B**) The bilateral penile skin was sutured to form a neourethra using 6-0 silk sutures without a testicular sheath. (**C**) The new urethra was reinforced and completely covered with the separated testicular sheath.

**Figure 3:**
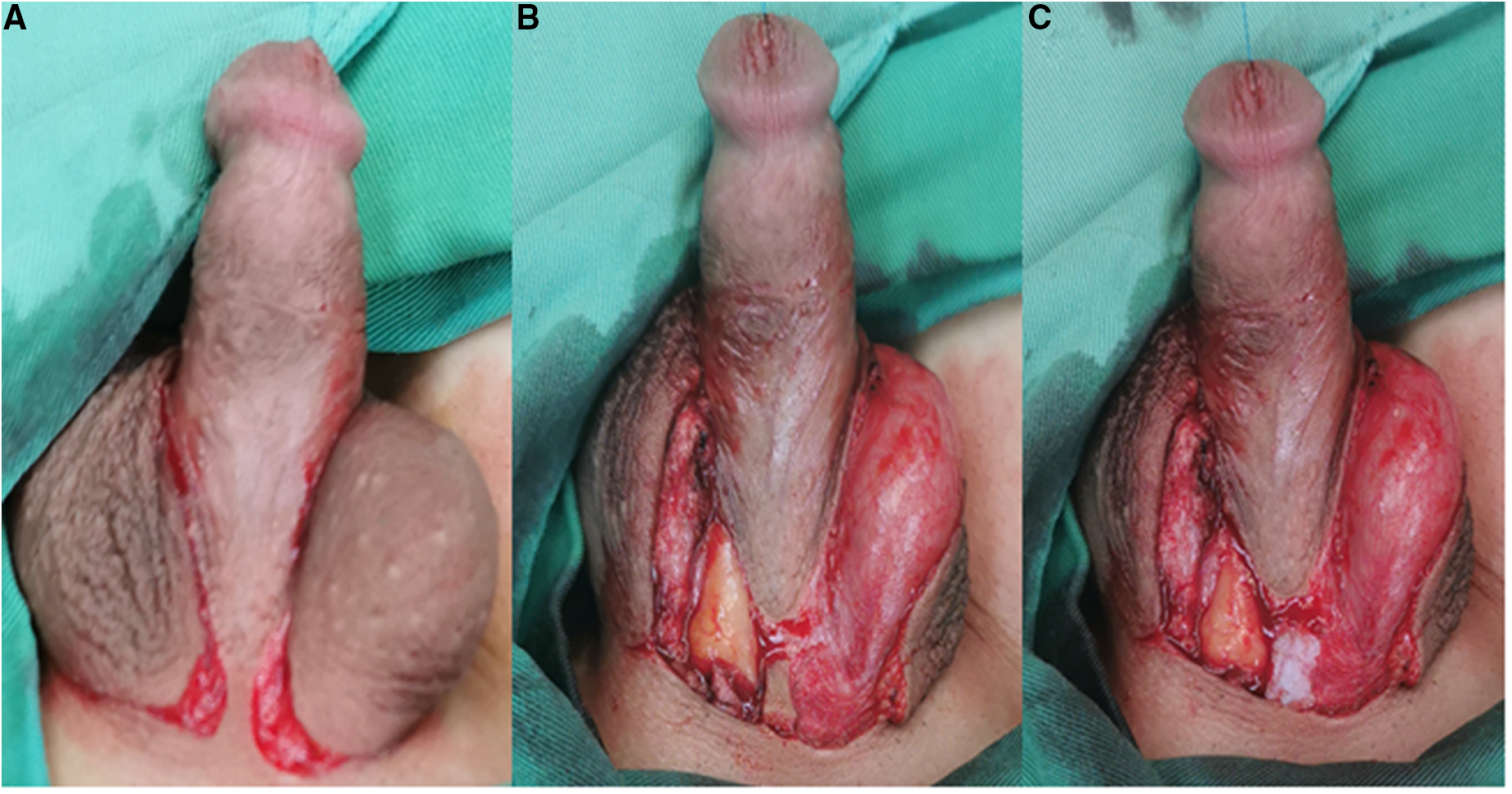
(**A**) An inverted “*Ω*” incision was made at the root of the penis. (**B**) The skin was removed. (**C**) The skin strip at the root of the penis was cut off.

**Figure 4:**
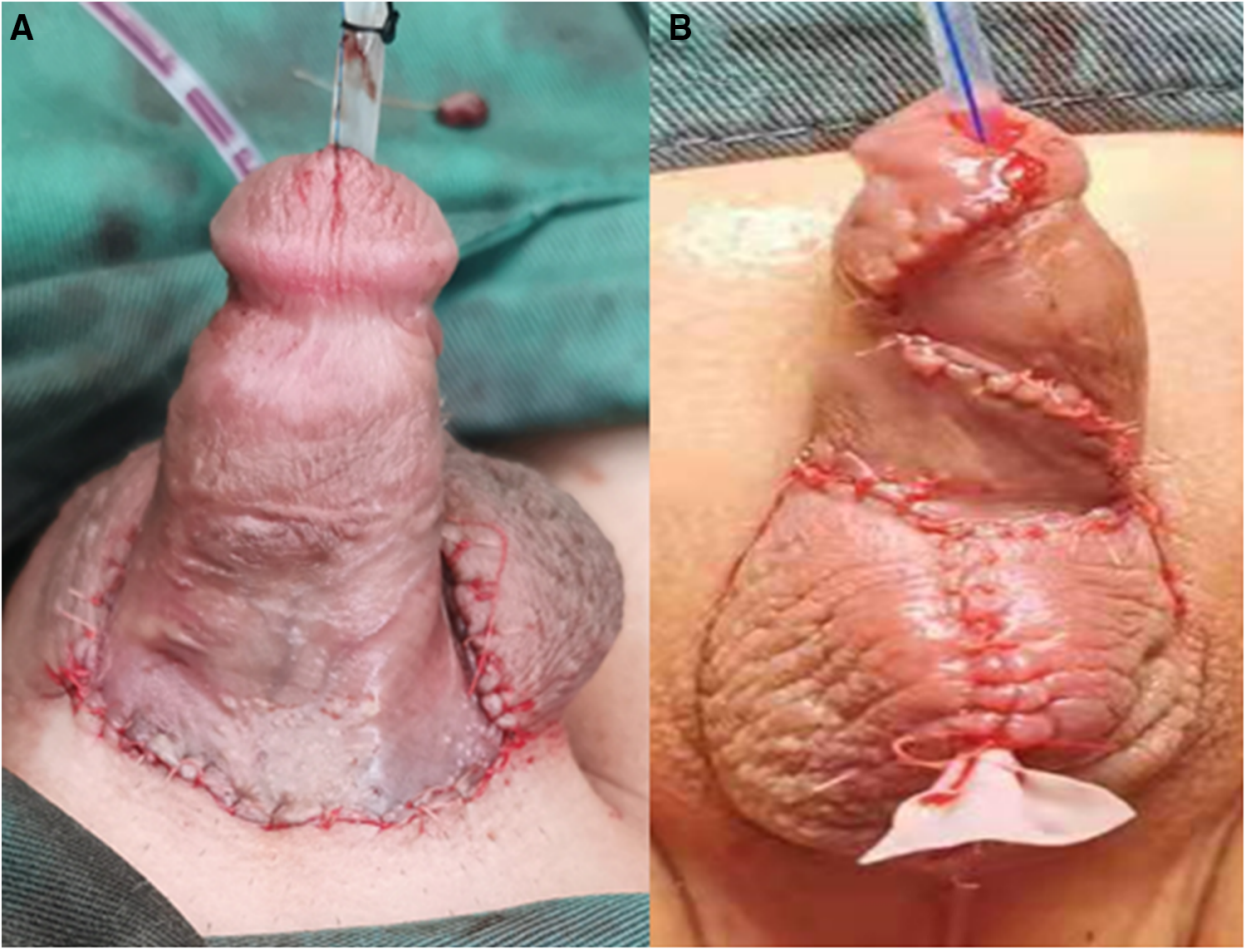
Reconstruction of penis and scrotum with flap transfer. (**A**) Dorsal view; (**B**) Ventral view.

The patient was transferred to a supine position. An incision was made around the root of the penis, the penis was stretched, and the meatus was moved away from the glans penis. Chordee was corrected as required. A U-shaped skin incision was cut with its base proximal to the urinary meatus and an 8F urethral catheter was inserted. The bilateral penile skin was sutured to form a neourethra using 6–0 silk sutures. The testicular tunica vaginalis was separated, with care to preserve the vascular pedicles under the skin. The new urethra was reinforced and covered with the separated testicular sheath (Figure 2). Next, an inverted “*Ω*” incision was made at the root of the penis, the skin was removed, and the skin strip at the root of the penis was cut off (Figure 3). Both the scrotal halves were dissected to the subcutaneous level, with care to preserve vascular pedicles; subsequently, the penis was lifted and the scrotum pulled down to correct transposition. The incision at the root of the penis was sutured. Two scrotal wings were thus created and mobilized by subcutaneous dissection; these two scrotal wings were rotated and sutured together. Finally, the penis and scrotum were reconstructed with flap transfer (Figure 4).

#### Group B

Urethroplasty was carried out in a similar way to Group A. The patient was transferred to a supine position. An incision was made around the root of the penis, the penis was stretched, and the meatus was moved away from the glans penis. Chordee was corrected as required. A U-shaped skin incision was cut with its base proximal to the urinary meatus and an 8F urethral catheter was inserted. The bilateral penile skin was sutured to form a neourethra using 6–0 silk sutures. The testicular tunica vaginalis was separated, with care to preserve the vascular pedicles under the skin. The new urethra was reinforced and covered with the separated testicular sheath. Next, the prepuce flap was dissociated, the double-winged flap was transferred to the ventral side of the penis, and the penis was wrapped and sutured with plastic surgery. After an interval of 3–6 months, the modified Glenn–Anderson technique was employed to repair incomplete penoscrotal transposition. An inverted “*Ω*” incision was made at the root of the penis, and the skin strip at the root of the penis was removed. Both the scrotal halves were dissected to the subcutaneous level, with care to preserve vascular pedicles; subsequently, the penis was lifted and the scrotum pulled down to correct transposition. The incision at the root of the penis was sutured. Two scrotal wings were thus created and mobilized by subcutaneous dissection; these two scrotal wings were rotated and sutured together. Finally, the penis and scrotum were reconstructed with flap transfer.

Highlights:
(1) We used the tunica vaginalis testis flap to repair the urethra.
(2) We removed the extra skin flap at the dorsal root at the time of modified Glenn–Anderson surgery.
(3) We combined the tunica vaginalis flap-covering Duplay technique with a modified Glenn–Anderson procedure in a one-step repair.

### Follow-up methods

After all stages of the surgery were complete, patients were followed up 1 month after urinary catheter removal and then at 3-month intervals thereafter for at least 1 year. Postoperative outcomes were evaluated primarily by ultrasound and by observation of the appearance of the penis and any complications.

Daniel et al.'s PPPS (pediatric penile perception score) (6) was used to evaluate perceived outcomes; specific items scored on this scale include the appearance of the shaft skin, general appearance, the configuration and appearance of the glans, and the configuration and position of the meatus. The patient's parents, the surgeon, and surgical peers were asked to express their satisfaction in relation to each item on a 4-point scale 3 months after the operation. The response options were: very dissatisfied (0 points), dissatisfied (1), satisfied (2), or very satisfied (3). The total PPPS was calculated by summing these scores for each of the items relating to the meatus, glans, shaft skin, and general appearance.

We also administered a questionnaire on urinary function. Meatal stenosis was defined as a meatal caliber of less than 8 Fr. We also judged whether urethral stricture was present using a symptoms questionnaire, which included questions on stranguria, dysuria, and urinary tract infection. Finally, we administered urination questions specific to hypospadias, including questions on whether the patient stands to urinate, whether they urinate from the end of their penis, whether they have more than one stream when they urinate (7), whether they experience spraying urine, whether their stream is straight (8), and whether they experience terminal dribbling (9). However, due to economic considerations and for medical insurance reasons, we did not use cystoscopy to diagnose urethral stricture.

### Statistical analysis

Data analysis was performed using SPSS 21.0. Mean values (±SD) are reported to describe patients' characteristics. All other variables are reported in the form of median values with the corresponding range. Student's *t*-tests were used to compare the groups, with *P* < 0.05 taken to denote statistical significance.

## Results

All patients underwent surgery for incomplete penoscrotal transposition with severe hypospadias. In all 86 cases, the severe proximal hypospadias and incomplete transposition were successfully repaired. No complications during the perioperative period (such as hematoma of the scrotum, recurrent curvature of the penis, urethral diverticulum, urethral dehiscence, wound infection, or flap necrosis) were observed in our study, and all patients were discharged within 7–11 days after surgery. There was no statistical difference between the two groups in terms of other complications (Table 1), nor was there any statistical difference between Group A operations and the first-stage operations for Group B in terms of operation time, postoperative off-bed time, or number of days of hospitalization. However, Group B patients also underwent a second-stage operation, with its attendant additional costs (Table 2). Three months after surgery, PPPS judgments (specifically, shaft skin and general appearance scores), as provided by parents and by the surgeon, were higher for Group A than for Group B. Most parents and surgeons were satisfied with the appearance of the genitals after one-stage repair (Table 3). Finally, 1 year after surgery, there was no statistical difference between the two groups in terms of parents' judgments on the urinary function questionnaire (Table 4).

**Table 1:** Complications occurring in the two groups.

Group	Stenosis of urinary meatus (%)	Urethral stricture (%)	Urinary fistula (%)	Urinary infection (%)
A (*n* = 42)	1 (2.38%)	1 (2.38%)	3 (7.14%)	3 (7.14%)
B (*n* = 44)	2 (4.54%)	1 (2.27%)	3 (6.82%)	2 (4.55%)
*P* value	0.279	0.948	0.907	0.309

**Table 2:** Operation time, postoperative off-bed time, and days of hospitalization in the two groups.

Group	Operation time (min)	Postoperative off-bed time (days)	Duration of hospitalization (days)	Cost (thousands)
A (one-stage operation) (*n* = 42)	63.21 ± 5.20	7.02 ± 1.32	10.55 ± 1.15	￥21.48 ± 2.64
B (first-stage/second-stage operation) (n = 44)	62.07 ± 4.47/50.59 ± 1.52	6.84 ± 1.20/6.16 ± 1.45	10.15 ± 1.45/9.16 ± 1.45	￥20.32 ± 2.49/19.05 ± 1.29
*P* value	0.059	0.456	0.092	0.869

There were no statistical differences between the groups at first-stage surgery in terms of operation time (*P* = 0.059), postoperative off-bed time (*P* = 0.456), days of hospitalization (*P* = 0.092), or cost (*P* = 0.869). Group B underwent a subsequent second-stage operation, incurring additional costs.

**Table 3:** Comparison of the two groups on PPPS, as judged by parents, the surgeon, and surgical peers 3 months after surgery.

PPPS item		Group A	Group B	*P* value
Meatus	Judged by parents	1.98 ± 0.75	1.91 ± 0.80	0.869
	Judged by surgeon	2.76 ± 0.57	2.79 ± 0.55	0.625
	Judged by surgical peers	2.36 ± 0.69	2.34 ± 0.81	0.217
Glans	Judged by parents	1.93 ± 0.87	1.93 ± 0.87	0.194
	Judged by surgeon	2.61 ± 0.53	2.63 ± 0.48	0.266
	Judged by surgical peers	2.05 ± 0.62	2.13 ± 0.55	0.776
Shaft skin	Judged by parents	2.10 ± 0.82	1.93 ± 0.62	0.028
	Judged by surgeon	2.42 ± 0.70	2.25 ± 0.58	0.025
	Judged by surgical peers	1.97 ± 0.60	2.02 ± 0.59	0.871
General appearance	Judged by parents	2.16 ± 0.91	1.93 ± 0.72	0.042
	Judged by surgeon	2.38 ± 0.69	2.29 ± 0.51	0.041
	Judged by surgical peers	2.16 ± 0.58	2.25 ± 0.49	0.694

**Table 4:** Comparison of the two groups on urinary function, as judged by parents.

Question	Yes/No	Group A *n* = 42 (%)	Group B *n* = 44 (%)	*P* value
Whether they stand to urinate	Yes	42 (100%)	43 (97.7%)	0.517
	No	0 (0%)	1 (2.3%)	
Whether they urinate from the end of their penis	Yes	42 (100%)	44 (100%)	1.000
	No	0 (0%)	0 (0%)	
Whether they have more than one stream when they urinate	Yes	1 (2.4%)	0 (0%)	0.488
	No	41 (97.6%)	44 (100%)	
Whether they experience spraying urine	Yes	5 (11.9%)	7 (15.9%)	0.412
	No	37 (88.1%)	37 (84.1%)	
Whether their stream is straight	Yes	39 (92.9%)	40 (90.9%)	0.526
	No	3 (7.1%)	4 (9.1%)	
Whether they experience terminal dribbling	Yes	2 (4.8%)	3 (6.8%)	0522
	No	40 (95.2%)	41 (93.2%)	

## Discussion

Penoscrotal transposition is a rare abnormality of the external genitalia, characterized by malposition of the scrotum superior to the penis (10). The condition is also known as prepenile scrotum or scrotopenile inversion (2). Patients with penoscrotal transposition may also present with other genital abnormalities, including hypospadias and chordee, as well as abnormalities of other organ systems (3). There are numerous possible approaches to surgical correction of proximal hypospadias following reconstruction of penoscrotal transposition, among which Thiersch–Duplay urethroplasty is one of the most common techniques (4). Severe proximal hypospadias is most often associated with incomplete penoscrotal transposition. Surgical treatment is based on the severity of the hypospadias and transposition (11). Both single-stage repair and multi-stage procedures for proximal hypospadias with incomplete penoscrotal transposition have been described in the literature (3, 12, 13). The complication rate for one-stage repairs, including Duckett repair, the Snodgrass procedure, and the Ehrlich and Scardino technique, is high because of the complicated nature of these repair procedures and a lack of blood supply (14). However, multi-stage repair usually means more pain and higher costs. To improve the viability of single-stage repair for proximal hypospadias and penoscrotal transposition, it is necessary to simplify the repair procedure and preserve the blood supply.

In our study, we probed a novel surgical method for proximal hypospadias with incomplete penoscrotal transposition. We have presented a new modified technique combining the Glenn–Anderson and the Duplay technique in a single stage to repair proximal hypospadias with penoscrotal transposition. To protect the blood supply, our method reinforces the newly-formed urethra with the pedicled tunica vaginalis of the testis. Additionally, in order to tighten the skin to achieve satisfaction in terms of appearance, we remove the extra skin flap at the dorsal root at the time of the modified Glenn–Anderson procedure. Both of the procedures are completed at the same stage. No complications during the perioperative period, such as urethral diverticulum, urethral dehiscence, wound infection, or flap necrosis, were observed in our study, and all patients were discharged within 7–11 days after surgery. There was no statistical difference between the groups undergoing single-stage repair and two-stage repair in terms of surgery time, complications, or duration of hospitalization. For patients and parents, the postoperative appearance of the penis is of paramount importance. The PPPS is a reliable instrument for urologist assessment and self-assessment of post-pubertal genitalia after hypospadias repair (15). In most cases, the parents, surgeon, and surgical peers were satisfied with the appearance of the genitals after one-stage repair.

There are few studies of severe hypospadias that have investigated the long-term outcomes of repair procedures using validated questionnaires. In the present study, we used a questionnaire to measure urinary function; this consisted of questions about urination specific to hypospadias, including whether the patient stands to urinate, whether they urinate from the end of their penis, whether they have more than one stream when they urinate (7), whether they experience spraying urine, whether their stream is straight (8), and whether they experience terminal dribbling (9). We also judged whether urethral stricture was present by asking about urination. The results indicated no statistical difference between the two groups on any of these variables. However, the general urinary function complication rate was lower than that occurring in other studies (7–9); the relatively short follow-up time is a possible reason for this. Measures of long-term outcomes need to be included to evaluate the therapeutic effects of this technique in future studies.

In some studies, uroflowmetry (volume, Qmax), endoscopy, and ultrasound for residual volume have been used to evaluate the outcome of hypospadias repair. However, as our center lacked sufficient equipment as a result of economic problems, we were unable to do so. Thus, more research is needed in the future to better understand the outcomes on these measures.

Some studies have described an alternative repair method for proximal hypospadias, in which urethroplasty is performed with the pedicled preputial skin flap, pedicled perimeatal connective tissue, pedicled scrotal fat, or a combination of these to protect the blood supply (16). However, limited tissue length often means that this tissue cannot extend down the entire length of the urethra. An unsatisfactory appearance and superficial skin necrosis are common outcomes (17). The original Koyanagi procedure might be one of the simplest and most effective methods for repair of proximal hypospadias with penoscrotal transposition. However, of 22 reported cases, three suffered from urinary fistula, one required re-operation because the external urethral orifice was retracted to the coronary sulcus of the penis, two showed recurrent curvature, and one showed slight stricture (18).

In contrast to the disadvantages of the abovementioned methods, the pedicled testicular tunica vaginalis can extend down the entire length of the urethra. Repair using the testicular tunica vaginalis is characterized by a thin, satisfactory appearance, elasticity, and an abundant blood supply. The tunica vaginalis flap could be an alternative to the preputial dartos fascia for covering the neourethra with a vascularized flap, resulting in fewer complications and acceptable results (19). The Glenn–Anderson technique is described as the correction of penoscrotal transposition *via* the design of rotational flaps that push the scrotum back while the penile skin remains attached by small strip to the skin of the mons pubis. However, chordee persists following this operation and sunken skin forms on the mons pubis, leading to an unsatisfactory appearance and wound infection. In the method described here, we removed the extra skin flap at the dorsal root, released the penis to avoid chordee, and eliminated the formation of sunken skin on the mons pubis to avoid wound infection. We also preserved the superficial dorsal artery and vein of the penis in order to protect the blood supply.

The limitations of our study include the fact that it was carried out at a single academic center and its retrospective nature, which makes it inherently subject to selection bias. Additionally, the follow-up period was relatively short. Measures of long-term outcomes need to be included to evaluate the therapeutic effects of this technique in future studies.

## Conclusion

In our study, we have demonstrated that the pedicled testicular tunica vaginalis flap can be used to cover the neourethra with a vascularized flap. For the purpose of preserving the superficial dorsal artery and vein of the penis to protect the blood supply, removal of the extra skin flap at the dorsal root was found to be safe. All of these procedures could be completed in a single stage, and produced acceptable results. However, long-term studies are required to support these conclusions.

## Data Availability

The original contributions presented in the study are included in the article/Supplementary Material, further inquiries can be directed to the corresponding author/s.
